# To Act or Not to Act: Endocannabinoid/Dopamine Interactions in Decision-Making

**DOI:** 10.3389/fnbeh.2015.00336

**Published:** 2015-12-17

**Authors:** Giovanni Hernandez, Joseph F. Cheer

**Affiliations:** ^1^Faculté de Pharmacie, Université de MontréalMontréal, Quebec, QC, Canada; ^2^Department of Anatomy and Neurobiology, University of Maryland School of MedicineBaltimore, Maryland, MD, USA; ^3^Department of Psychiatry, University of Maryland School of MedicineBaltimore, Maryland, MD, USA

**Keywords:** endocannabinoids, dopamine, pre-clinical studies, decision-making, reward

## Abstract

Decision-making is an ethologically adaptive construct that is impaired in multiple psychiatric disorders. Activity within the mesocorticolimbic dopamine system has been traditionally associated with decision-making. The endocannabinoid system through its actions on inhibitory and excitatory synapses modulates dopamine activity and decision-making. The aim of this brief review is to present a synopsis of available data obtained when the endocannabinoid system is manipulated and dopamine activity recorded. To this end, we review research using different behavioral paradigms to provide further insight into how this ubiquitous signaling system biases dopamine-related behaviors to regulate decision-making.

## Scope and Introduction

When presented with several alternatives and when deciding which course of action to take, organisms have to integrate different pieces of information. These pieces of information include, but are not limited to the size of the reward, risk, physiological states and expected time to obtain a reward. After the integration of these pieces of information, it is anticipated that the organism will choose the option with the highest value. Given the combination of different variables at the moment of making a decision, the value of the reward rarely represents its objective properties, it represents subjective desirabilities. The representation of idiosyncratic values of different goal objects is encoded in different neural systems (Kable and Glimcher, 2007). Numerous studies on decision-making have shown that dopamine (DA) plays a critical role in the representation of multiple variables underlying the perceived reward value and reward-seeking. This is not surprising, given the array of structures to which DA neurons project and the different modalities of DA release. For some researchers, DA phasic firing encodes reward value (Tobler et al., 2005; Kobayashi and Schultz, 2008). Others have emphasized the role of DA release in different brain areas in the modulation of different processes involved in reward valuation. For example, in the modulation of reward sensitivity (Wise and Rompre, 1989) or reward-gain (Hernandez et al., 2010; Hernandez and Cheer, 2012), in the modulation of effort cost and vigor (Niv et al., 2007; Salamone et al., 2007); whereas others have put an emphasis on the role of DA release in incentive salience (Berridge and Robinson, 1998).

Given the profusion of processes that DA firing and release is involved, it is of great interest to understand how other brain networks alter the DAergic system activity. One system that is pivotal in the modulation of different circuitries is the endocannabinoid (eCB) system. The eCB system, through its interaction with excitatory and inhibitory afferents to the ventral tegmental area (VTA), has proven to be critical in fine-tuning decision-making processes (Melis and Pistis, 2012). Here, we will review the effects of activation and inactivation of eCBs on diverse appetitive behavioral paradigms and how these manipulations alter the behavior and accompanying DA dynamics.

## Brief Introduction to the eCB System

Ever since the cloning of the central cannabinoid receptor CB1R (Matsuda et al., 1990) and the isolation of the first endogenous cannabinoid (eCB) (Devane et al., 1992), the eCB system has been of great interest to neuroscientists. CB1Rs are the most abundant G_i/o_ coupled receptors found in the brain (Herkenham et al., 1991; Howlett et al., 2002) and they modulate a wide array of functions and processes, ranging from motor control to decision-making.

The eCB system is comprised of cannabinoid receptors (CB1R, CB2R), their endogenous ligands and the enzymes that degrade them. The ligands most thoroughly studied and characterized are anandamide and 2-arachidonoyl glycerol (2-AG). eCBs are lipid-derived metabolites that are produced “on-demand” by postsynaptic cells and immediate released. The signal that the cell uses to start the biochemical cascade for eCBs synthesis is, in general, an enhancement in intracellular Ca^2+^ concentration. This increase in Ca^2+^ is due to cell depolarization or mobilization of intracellular Ca^2+^ stores (for a detailed review on the synthesis of eCBs; see Di Marzo, 2006). Once produced and released, eCBs act retrogradely mostly onto CB1R localized on excitatory (glutamatergic) and inhibitory (GABAergic) terminals (Elphick and Egertová, 2001; Wilson and Nicoll, 2001; Alger, 2002). The activation of these receptors produces molecular changes leading to the closing of Ca^2+^ (N- and P/Q type) channels (Twitchell et al., 1997) and/or opening of K^+^ channels (Mackie et al., 1995). The effect of these changes at the cellular level is to reduce the probability of neurotransmitter release (Maejima et al., 2001; Lupica and Riegel, 2005) thus, influencing both short- and long-term forms of synaptic plasticity (Alger, 2002). After eCBs reach their target, they are rapidly degraded. Specifically, fatty acid amide hydrolase (FAAH) participates in anandamide degradation (Di Marzo et al., 1994) whereas monoacylglycerol lipase (MAGL) degrades 2-AG (Dinh et al., 2002; De Petrocellis et al., 2004).

## eCB Actions in the Mesocorticolimbic Reward System

eCBs modulate decision-making in part by curbing the activity of excitatory and inhibitory neurotransmission along the mesocorticolimbic pathway. eCBs are an important neural substrate involved in decision-making processes (Wise and Rompre, 1989; Koob, 1992; Chao and Nestler, 2004) and in the processing of rewarding stimuli (Wise and Rompre, 1989; Salamone and Correa, 2002; Schultz, 2010; Hernandez et al., 2012). eCB activation of CB1Rs at the level of the ventral tegmental area (VTA), the site of origin of the mesocorticolimbic dopaminergic (DA) neurons, increases DA burst firing (French, 1997; Gessa et al., 1998; Wu and French, 2000). As consequence, it facilitates DA release in terminal areas such as the nucleus accumbens (NAc) and the prefrontal cortex (Chen et al., 1990; Pennartz et al., 1994; Tanda et al., 1997; Cheer et al., 2007a; Oleson et al., 2012). Such change in DA excitability is significant because empirical evidence implicates these neurons in the encoding of the subjective value of the reward (Tobler et al., 2005; Roesch et al., 2007; Kobayashi and Schultz, 2008; Roesch and Bryden, 2011; Lak et al., 2014). When the size or delay of a reward are manipulated, DA neurons fire at a higher rate for the cues that predict the subjective more valuable reward (i.e., larger reward or shorter delay; Roesch et al., 2007). When effort and delay to obtain a reward are manipulated, phasic DA release in the NAc is higher at the cue that predicts lesser exertion. Phasic DA release also increases at cues that predict an immediate reward (Day et al., 2011). Importantly when DA signaling is disrupted, changes in the behavior ensue so that subjects no longer adapt their behavior according to changes in reward contingencies (Cardinal et al., 2001; Ghods-Sharifi and Floresco, 2010; Stopper et al., 2014).

Given that DA neurons do not express CB1Rs (Herkenham et al., 1991; Matsuda et al., 1993; Julian et al., 2003), the modulation of their activity and release by eCBs comes indirectly from the activation of CB1R present on afferents to DA cell bodies. Under conditions of relatively high neural activity, DA neurons release eCBs (Alger, 2002; Melis et al., 2004). These molecules retrogradely bind to CB1R on presynaptic terminals to dampen the activity of DA afferents. This reduction in the activity of DA inputs allows DA neurons to regulate their activity levels (Melis et al., 2004, 2006; Marinelli et al., 2007). The precise mechanism by which eCBs facilitate DA burst firing in a behaving animal remains to be fully established. One possibility is that DA burst firing is the result of the net effect of eCBs on the combined probabilities of glutamate and GABA release (Lupica and Riegel, 2005; Melis and Pistis, 2007). Under normal resting circumstances, approximately 50% of DA neurons are under inhibitory GABAergic drive (Grace and Bunney, 1984) rendering them insensitive to excitatory inputs. Direct activation of CB1R on GABA neurons reduces inhibitory drive on DA neurons, making them more susceptible to excitatory inputs and therefore, more prone to fire in bursts (Overton and Clark, 1997; Zweifel et al., 2009). Nonetheless, activation of CB1R on glutamatergic neurons also reduces the probability of glutamate release. This reduction would have a dual effect; it would diminish the excitatory inputs to DA neurons (Melis et al., 2004), which would curtail burst firing, but it could also reduce GABAergic inhibitory drive onto DA neurons. Indeed, glutamate plays a major role in the maintenance of DA inhibitory drive by acting on NMDA receptors located in GABA neurons most likely through GluN2A receptors (Bergeron and Rompré, 2013; Hernandez et al., 2015). A reduction in glutamate release probability, therefore, adds to an overall decrease in DA inhibitory drive. Although eCBs can lower the probability of glutamate release, the effect is limited due to the greater relative presence of CB1R on GABAergic vs. glutamatergic terminals (Mackie, 2005). The combined effect of decreased glutamate release and CB1R-induced activation of GABA neurons would result in a net reduction in the number of DA neurons firing in a slow tonic manner. Under these conditions, DA neurons are ready to fire in bursts once NMDA receptors are activated (Overton and Clark, 1997; Zweifel et al., 2009; but see Lobb et al., 2010 for an alternative mechanism).

eCB-induced disinhibition of DA neurons in the VTA can be produced intrinsically by acting on GABAergic interneurons or extrinsically via GABAergic afferents (Lupica et al., 2004). This distinction is possible by the general type of GABA receptor involved. GABAergic interneurons preferentially target GABA_A_ receptors located on VTA DA neurons; whereas GABAergic afferents target preferentially GABA_B_ receptors (Johnson and North, 1992; Sugita et al., 1992). *In vitro* experiments show that the excitatory effect of the CB1R agonist HU-210 is occluded by application of the GABA_A_ receptor antagonist bicuculline or the CB1R antagonist rimonabant to the slice (Cheer et al., 2000). Similarly, perfusion of the CB1R agonist WIN55, 212–2 decreases electrically evoked inhibitory postsynaptic currents (IPSCs) in a GABA_A_ receptor-dependent manner (Szabo et al., 2002); whereas application of the CB1R antagonist rimonabant prevents this effect. In addition to this intrinsic mechanism for the eCB dependent disinhibition of VTA DA neurons, an extrinsic disinhibition mechanism has been hypothesized which acts predominantly on GABA afferents targeting GABA_B_ receptors (Riegel and Lupica, 2004). Here, the application of CB1R agonist WIN55, 212–2 decreases the amplitude of the GABA_B_ mediated IPSCs, in a CB1R-dependent fashion. However, immunocytochemical investigations have not yet identified the origin of such VTA GABA afferents (Mátyás et al., 2008). Further electrophysiological research points towards the: (a) NAc, a critical brain area mediating appetitive behaviors via the integration of inputs from cortical and limbic structures (Mogenson et al., 1980); (b) ventral pallidum, a region that plays a part in the differentiation of wanting, liking, and prediction components of a reward (Smith et al., 2011); and (c) rostromedial tegmental nucleus (RMTg), a small node that plays a pivotal role in processing both aversive and appetitive stimuli (Jhou et al., 2009b).

The projection of medium spiny neurons (MSN) of the NAc to the VTA was one of the first afferents proposed (Walaas and Fonnum, 1980; Sugita et al., 1992; Kalivas et al., 1993). It was hypothesized that these axon terminals converged onto DA neurons and directly inhibited DA activity (Einhorn et al., 1988; Rahman and McBride, 2000). However, recent evidence using genetic and optogenetics tools is at odds with this notion. Optical activation of NAc MSN demonstrated that these axons mainly synapse onto non-DA neurons, and these connections are fast–inhibitory neurons mediated by GABA_A_ receptors (Xia et al., 2011). Moreover, it was demonstrated that CB1 expressing neurons in the NAc are fast-spiking interneurons, not MSNs. A conclusion obtained via the use of a knock-in mouse line in which CB1-expressing neurons also expressed the fluorescent protein td-Tomato (Winters et al., 2012). These results imply that synaptic projections from the NAc to the VTA should not be affected by CB1R signaling, although further research utilizing more sophisticated retrograde labeling techniques is needed.

*In vivo* electrophysiological studies show that GABA projections coming from the VP (Aguilar et al., 2015) and RMTg (Lecca et al., 2011, 2012) are sensitive to cannabinoid manipulations, and they modulate VTA DA neural firing. Inhibiting the degradation of eCBs in the VP decreased VTA DA neural activity observed following chronic treatment with the NMDA glutamate receptor antagonist phencyclidine (Aguilar et al., 2015). Likewise, manipulation of the RMTg nucleus has a profound effect on DA neural firing. The RMTg receives dense, mostly glutamatergic inputs from the lateral habenula (Jhou et al., 2009a,b), an area that encodes aversive stimulation (Matsumoto and Hikosaka, 2009). This nucleus mediates the inhibitory effect of the lateral habenula on midbrain DA neurons (Jhou et al., 2009a,b). The RMTg neurons that project to the VTA form inhibitory synapses, so that activation of this input, via electrical stimulation, inhibits DA firing (Lecca et al., 2011). Systemic injections of CB1R agonist produces a long-lasting decrease in the firing rate of GABA neurons located in the RMTg. The administration of a CB1R antagonist, which on its own is devoid of effects on firing rate of GABA neurons, minutes before the agonist, prevents the inhibition of RMTg GABA neurons. *In vitro* recordings, demonstrate that the reduction in the amplitude of excitatory postsynaptic currents is the mechanism underlying the inhibition of GABA neurons. In addition to a decrease in excitatory postsynaptic currents, CB1R agonist produced a significant increase in paired-pulse ratio, suggesting that the CB1R agonist produced a reduction in glutamate release through activation of presynaptic receptors (Lecca et al., 2011). As expected, the inhibition of GABA neurons in the RMTg correlates with an increase in firing of DA neurons in the VTA (Lecca et al., 2011, 2012).

These electrophysiological studies suggest that eCB modulation of afferents to the VTA potently regulate DA activity via multiple mechanisms. The modulation of DA responses has important implications for decision-making processes. If, by their phasic firing and release, DA neurons integrate the subjective reward value (Lak et al., 2014) then eCB signaling is crucial during reward evaluation and can alter the weight of the variables used during goal assessment. Once different alternatives are weighted, and different goals are assessed, subjects have to start an action according to their assessment; such course of action is believed to represent the option with the highest expected subjective preference. Thus, reward-seeking can be used as a proxy to infer the subjective reward value and changes in decision-making. In the following section, we will review empirical evidence that shows how altering DA signaling via CB1R manipulations biases goal-directed behavior.

## eCBs and BSR

Several organisms will deliver electrical pulse trains to different brain areas via insulated macro electrodes (Olds and Milner, 1954; Olds, 1962; Shizgal and Murray, 1989). The effect of the electrical stimulation that leads organisms to seek and reinitiate the stimulation is called brain stimulation reward (BSR). Since its discovery, BSR has become the paradigm of choice for studying the neural reward circuitry and goal-directed responses. The rewarding signal that arises as a result of the delivery of electrical pulses shares properties with natural rewards. BSR can compete with, summate with natural rewards (Conover and Shizgal, 1994) and BSR can be degraded in a similar way as natural rewards (Hernandez et al., 2011). These characteristics strongly suggest that the behavior maintained by pulses of electrical brain stimulation is far from being rigid or habitual responding (Hernandez et al., 2011), but denotes the subject’s integration of different pieces of information regarding the value of different outcomes. During intracranial self-stimulation (ICSS), the experimental subject has to choose between pressing the lever to trigger electrical pulses or engage in competitive activities, i.e., exploring the box, sniffing or resting. The time allocated to each activity by the experimental subject will depend on the perceived value of the stimulation.

Can the reward induced by the electrical pulses change by altering DA neurotransmission? ICSS was the first paradigm implemented to study different reward substrates and the role of DA in reward (Crow, 1972a,b). Indeed, the electrical train pulses injected by the electrode produce an increase in DA cell firing and DA release (Moisan and Rompré, 1998; Hernández and Shizgal, 2009). A large body of evidence shows that reward induced by electrical brain stimulation is highly sensitive to changes in VTA DA neurotransmission, as measured by the curve-shift paradigm. In this experimental preparation, a series of stimulation parameters (pulse frequencies or currents) that drives response rate from a maximal to a minimal level in an S-shaped manner are used (Miliaressis et al., 1986). Drugs that enhance DA levels such as DA transporter blocker GBR12909 produce a leftward displacement of the curve that relates operant performance to stimulation parameters (Rompré and Bauco, 1990). Thus, it is inferred that these drugs boost the rewarding effect of electrical brain stimulation. Opposite effects are obtained with DA receptor antagonists like haloperidol and raclopride (Nakajima and Baker, 1989).

What are the consequences of manipulating CB1Rs on ICSS? Since CB1R agonists increase DA output (Ng Cheong Ton et al., 1988; Chen et al., 1990); it was expected that they would potentiate the rewarding signal that arises from the electrical stimulation; whereas CB1 receptor antagonists would do the opposite. However, research from different groups yielded inconsistent evidence. The first reports using Δ^9^-THC, showed a reward enhancement effect that was dependent on the rat strain, such differences in rat strain correlated with differences in DA efflux in the NAc. Lewis rats showed the larger behavioral effect as well as, the higher DA release following the administration of Δ^9^-THC. In contrast, Fisher and Sprague-Daley rats showed a minimal behavioral effect and modest DA increments (Chen et al., 1991; Lepore et al., 1996). Several other studies using Long-Evans or Sprague-Daley rats have found a decrease in reward pursuit or no effect (Stark and Dews, 1980; Vlachou et al., 2007); whereas others have found different results depending on the dosage of Δ^9^-THC used. At low doses (0.1 mg/kg) a facilitation on reward is seen; whereas at a higher doses (1 mg/kg) a hindrance on reward is obtained (Katsidoni et al., 2013). Similar puzzling effects were observed with other CB1R agonists (Arnold et al., 2001; Antoniou et al., 2005). Using indirect agonists such as inhibitors of the enzymes that degrade eCBs (Vlachou et al., 2006; Kwilasz et al., 2014), has yielded a lack of effect or a decrease in reward pursuit (Arnold et al., 2001; Deroche-Gamonet et al., 2001; Vlachou et al., 2006).

These disparate results obtained in ICSS experiments using the curve-shift paradigm could be due to genetic differences as Gardner’s experiments suggest (Chen et al., 1991). Another explanation could be that systemic injections of these compounds produce an indiscriminate activation of all brain areas containing CB1Rs. Given that CB1Rs are the most abundant G-protein-coupled receptors in the brain (Herkenham et al., 1990) such broad activation is problematic for studying the neural underpinnings of reward evaluation and reward-seeking. These processes most likely require the activation of eCB synthesis and release to be region, neuron or even synapse-specific (Solinas et al., 2008). Thus, a wide activation of CB1R might give rise to negative or dysphoric effects that counteract their positive action on reward-seeking (Panagis et al., 2014). However, these explanations do not resolve why when using other experimental testing procedures (i.e., progressive ratio) CB1R agonist and antagonist produce behaviorally consistent results, even when using systematic injection and dose ranges similar to the ones used in ICSS experiments.

An alternative possibility relies on findings that the effects of CB1R agonists on DA release in the NAc are moderate at best when contrasted with other DA agonist or DA receptor blockers. Such modest DA release is problematic for traditional curve-shift paradigms used in ICSS experiments. The curve-shift paradigm lacks the dimensionality to differentiate between changes in the relative reward strength, the only dimension measured in this experimental preparation from changes in costs (opportunity and effort), to obtain a goal object. All these variables contribute to goal evaluation, and different researchers have shown the modulation of these by changes in DA efflux (Wise and Rompre, 1989; Salamone and Correa, 2002; Hernandez et al., 2012). So when using a two-dimensional perspective, non-measured changes on the “hidden” dimension can be misconstrued as an effect the subjective reward intensity.

Why is this methodological distinction important? If DA release does not modulate the relative value of a reward, then moderate changes in DA release would produce unreliable changes in curves relating behavior and stimulation intensity; as it is the case with CBRs agonist. When using the “mountain-model” (Arvanitogiannis and Shizgal, 2008) a testing paradigm that measures opportunity cost in addition to changes in stimulation strength, CB1R antagonists produce consistent decreases in opportunity cost. This reduction correlates with a consistent decrease in DA release (Trujillo-Pisanty et al., 2011). This effects mimics that of DA receptor antagonists (Trujillo-Pisanty et al., 2013), and it is consistent but of opposite direction with results obtained with non-specific and specific DA transport blockers (Hernandez et al., 2010; Hernandez and Cheer, 2012).

## eCBs and Motivation

When electrical stimulation is used to study the effects of different manipulations on the eCB system, the results appear contradictory. With the inclusion of a third variable in the measuring paradigm, these are reconciled with the rest of the scientific research implicating CB1Rs in reward modulation, relating the motivation for obtaining different classes of rewards evaluated by several schedules of reinforcement. One of these is the progressive ratio where the requirements to acquire a single reward is exponentially increased, within a single session until the experimental organism stops responding. “Breakpoint” is the ratio at which the subject stops responding. It is assumed that this schedule measures the relation between response effort and the value of a particular reward (Hodos, 1961). Thus, inferences about the willingness of the organism to work to obtain a goal object can be drawn.

If cannabinoid agonists are used in conjunction with this schedule, they increase breakpoints. Thus, the experimental subjects are willing to lever-press more for a single reward. This effect has been consistent across different classes of rewards (Higgs et al., 2005; Solinas and Goldberg, 2005; Ward and Dykstra, 2005; Gamaleddin et al., 2012; Jones and Kirkham, 2012; Oleson et al., 2012) and equally consistent but opposite effects have been obtained with CB1R antagonists (Solinas and Goldberg, 2005; Ward and Dykstra, 2005; Maccioni et al., 2008; Rasmussen and Huskinson, 2008; Xi et al., 2008; Gamaleddin et al., 2012; Hernandez and Cheer, 2012). Recent research shows that inhibiting 2-AG degradation, but not anandamide increases breakpoints. Moreover, intra-VTA inhibition of 2-AG degradation facilitates reward-seeking and DA phasic release (Oleson et al., 2012).

## eCBs and Discounting

The value of a goal object depends on how distant in the future it is. When an organism is deciding among different goal objects, it has to consider into its computations how distant in the future different goals objects are and take a decision based on the best-perceived alternative. At the decision point, the organism will select the option with the highest perceived value. Temporal discounting can be measured by allowing experimental subjects to choose between two alternatives: one that delivers an immediate but small reward vs. another that delivers a larger but delayed reward. Under this arrangement, and when questioned about future choices humans and non-humans subjects show a preference for the larger distant reward over the immediate small one. However, as time passes the difference between the small and large reward becomes less prominent and preference switches (Ainslie, 1975), this change occurs because immediate rewarding outcomes have a greater subjective value than delayed ones. Self-control is exercised when the delayed option is still preferred, whereas impulsivity takes place if the immediate option is chosen (Rachlin and Green, 1972).

DA firing and release are critically important for temporal discounting. DA phasic firing correlates positively with the magnitude of reward and decreases in a hyperbolic fashion with reward delay (Kobayashi and Schultz, 2008). Similarly, DA release in the NAc shows patterned release at the cue that predicts different delays. It shows a decrease that correlates with the length of the delay (Saddoris et al., 2015). When phasic DA release is measured at reward delivery DA release is higher for the larger reward at small to moderate delays, and then decreases to a level comparable to that of the small immediate reward (Hernandez et al., 2014).

By pharmacologically manipulating the DA system, during intertemporal choice tasks, different studies have shown that acute challenges with drugs that increase DA availability produce an increase in self-control. Experimental subjects choose more often the large delayed reward over the immediate small one (Cardinal et al., 2000; Wade et al., 2000; Winstanley et al., 2003, 2005; van Gaalen et al., 2006; Bizot et al., 2007). Conversely, drugs that interfere with DA availability produce an increase in impulsive choice (Wade et al., 2000; van Gaalen et al., 2006; Floresco et al., 2008). Experimental subjects choose more often the small immediate reward over one large delayed one. As a modulator of the DA system, eCB signaling produces remarkable results. Acute activation of CB1Rs with Δ^9^-THC leads to increased self-control that is blocked by CB1R antagonists. Interestingly, CB1R antagonists attenuate the effect of DA agonists (Wiskerke et al., 2011). When given alone CB1R antagonists do not exert a significant influence on self-control. These results suggest that the eCB system does not play a role in baseline temporal discounting (Pattij et al., 2007; Wiskerke et al., 2011; Hernandez et al., 2014).

Although acute increases in DA release increase self-control, the opposite happens when subjects have chronic experience with different drugs of abuse that directly or indirectly alters DA neurotransmission (Di Chiara and Imperato, 1988). Chronic drug exposure produces plastic changes in the mesolimbic circuitry and other brain areas and neurotransmitters that underlie addiction (Nestler, 2001; Everitt and Robbins, 2005; Kalivas and Volkow, 2005). As stated above, eCBs participate in the modulation of synaptic plasticity in the VTA (Melis et al., 2004; Haj-Dahmane and Shen, 2010); where they modulate DA neuron excitability (Lupica and Riegel, 2005; Maldonado et al., 2006). eCBs play a critical role in the increase of phasic DA release observed after the administration of different types of drugs of abuse (Cheer et al., 2007b). They are necessary for the development and expression of sensitization (Viganò et al., 2004; Corbillé et al., 2007; Azizi et al., 2009; Li et al., 2009; Blanco et al., 2014; Mereu et al., 2015). Also, eCB signaling is required for conditioned drug seeking and relapse (De Vries et al., 2001; De Vries and Schoffelmeer, 2005; Maldonado et al., 2006) as well as cue-induced reinstatement (De Vries et al., 2001, 2003; Xi et al., 2006). Therefore, the eCB system is likely to play a role in impulsive behavior observed in drug addiction.

Our laboratory recently found that eCB signaling is a canonical component in the development of impulsive choice caused by chronic cocaine exposure. Specifically, after experimental subjects were sensitized to the effects of cocaine they behaved impulsively in an intertemporal choice task (Mendez et al., 2010; Hernandez et al., 2014; Smethells and Carroll, 2015). The pattern of DA release in the NAc during the task correlates with behavioral performance. Before sensitization, higher DA release is observed for the larger reward when the delay is below 10-s. After sensitization has taken place, phasic release for the small immediate reward is comparatively higher regardless of the delay. Importantly, blockade of CB1Rs before cocaine exposure prevented not only impulsive choice, but it also eliminated maladaptive patterns of phasic DA release. More importantly, from a therapeutic perspective, CB1R blockade reverted changes in self-control observed following cocaine sensitization (Hernandez et al., 2014).

## Future Directions

The research showcased in the present review demonstrates that the eCB system, via modulation of phasic DA release, plays important roles in decision-making processes. eCB signaling is critical for adjudicating value to different rewards as well as for activating, organizing and maintaining goal-directed behaviors. This happens under normal circumstances and is usurped when decision-making processes are compromised. However, the current state of this body of research is only a first step that will lead to a better understanding of the potential reach of the eCB system in decision-making processes. To further our knowledge, it is important to map each eCB action in all of the relevant circuits thoroughly. This requires elucidating the exact localization of CB1 receptors and their active ligands on cell-type specific nodes and under temporally-resolved circumstances. Such a targeted approach will greatly enhance our current understanding of the anatomical frameworks engaged in decision-making processes. With this information in hand, it will be possible to create models that more accurately predict changes in the behavior and the underlying neurochemistry.

## Conflict of Interest Statement

The authors declare that the research was conducted in the absence of any commercial or financial relationships that could be construed as a potential conflict of interest.
